# Evaluation of therapeutic effect of targeting nanobubbles conjugated with NET-1 siRNA by shear wave elastography: an *in vivo* study of hepatocellular carcinoma bearing mice model

**DOI:** 10.1080/10717544.2019.1667450

**Published:** 2019-09-23

**Authors:** Haitao Shang, Bolin Wu, Xitian Liang, Yixin Sun, Xue Han, Lei Zhang, Qiucheng Wang, Wen Cheng

**Affiliations:** Department of Ultrasound, Harbin Medical University Cancer Hospital, Harbin, China

**Keywords:** Shear wave elastography, targeted nanobubbles, gene therapy, nude mice with hepatocellular carcinoma, low-frequency ultrasound

## Abstract

This study aimed at investigating the tumor stiffness of hepatocellular carcinoma (HCC) bearing mice model in vivo to evaluate the therapeutic efficacy of targeting nanobubbles (TNBS) conjugated with NET-1 siRNA (NET-1 siRNA-TNBS). Also tested whether shear wave elastography (SWE) could demonstrate the pathological tumor changes and used to monitor therapeutic efficacy as a noninvasive method. The HCC bearing mice model was established by injecting human HCC cell line (HepG2). The mice were then divided into three groups randomly, and were treated with TNBS conjugated with NET-1 siRNA, TNBS conjugated with negative control gene, and saline as control. US-SWE was performed for three times. SWE values of all the tumors in three groups were increased with tumor growth. Emax was correlated with tumor size (*p* < .05). NET-1 gene (treatment group) significantly delayed the growth of tumor size compared to other two groups (*p* < .0001), showing a significantly increased Emax (*p* < .05). Immunohistochemical results showed that the NET-1 protein expression was significantly lower than the negative control and blank groups. In conclusion, TNBS conjugated with NET-1 siRNA inhibited tumor growth and prolonged the life of experimental animals. SWE provided a noninvasive and real time imaging method to detect the changes in tumor development.

## Introduction

Hepatocellular carcinoma (HCC) is one of the most common cancers in the world and has several genetic alterations (Forner et al., 2014). There are various therapeutic methods for the treatment of HCC, but are limited due to high recurrence and rapid progression (Bruix et al., 2014). Thus, novel effective agents to effectively suppress HCC are imperative (Li et al., 2016). Many research studies have suggested that gene therapy can delay the development of cancer (Das et al., 2015; Kumar et al., 2016; Baruteau et al., 2017), and accordingly RNA interference (RNAi) technology has been rapidly developed in clinical trials (Davidson & Mccray, 2011). It has been shown that the New EST tetraspanin-1, also called as NET-1 (C4.8, Tspan-1, P503S), overexpressed in various human tumor tissues (Zhang et al., 2014). This was strongly associated with pathological grading and clinical stages of HCC (Chen et al., 2007), making NET-1 as a potential therapeutic target for HCC. Recently, microbubbles have been widely used as tools for gene delivery in the target tissues because of their visibility (Geis et al., 2012), and the shell of microbubbles could protect siRNA against nucleas, but these can penetrate through large endothelial cell space in tumor vessels (Ferrara et al., 2007). Therefore, researchers have found that nanobubbles are gene delivery tools that pass through the endothelial cell space and penetrate the interstitial space of tumor samples more easily. In the process of study, microbubbles were scattered by low-frequency ultrasound irradiation, the growth, formation, oscillation and collapse of microbubbles were referred as acoustic cavitation, stable cavitation cause local streaming and increase the permeability of blood vessels, furthermore, cavitation produce fewer adverse effects in the tissues, so it can be a promising method of gene delivery. To enhance the transfection effect of NET-1 siRNA delivery, we prepared targeting nanobubbles (TNBS) conjugated with NET-1 siRNA successfully and also verified the effectiveness in vitro by combining NET-1 siRNA TNBS and low-frequency ultrasound (Wu et al., 2016).

The stiffness of lesion could be an parameter for assessment of treated tumor tissue, which could be evaluated with ultrasound, because response to therapy is associated with the changes in the biomechanical properties of cancer (El Kaffas et al., 2013). Shear wave elastography (SWE) is a quantitative technique which has been widely used in liver fibrosis and liver tumors (Piscaglia et al., 2014). Recently, some studies have demonstrated the effect of special treatment in mice tumor models by using SWE (Chamming’s et al., 2013; Li et al., 2014), but few focused on the changes of tumor hardness caused by gene therapy. Therefore, we used SWE to explore the potential changes in tumors of HCC bearing mice model, and evaluate the therapeutic effect of TNBS conjugated with NET-1 siRNA in the present study.

## Materials and methods

### Preparation of nanobubbles and siRNA-conjugated TNBS

The lipid nanobubbles obtained from liposomes were prepared from DSPC, DSPE-PEG-2000 and DSPE-PEG2000-biotin (purchased from Avanti Polar Lipids, Alabaster, AL) at a molar ratio of 9:0.5:0.5. These were dissolved in chloroform and were blended. The chloroform was then removed at 37 °C using vacuum rotary evaporation. Next, we evaporated the phospholipid mixture to dryness, and were dried at 40 °C with 5 mL phosphate-buffered saline (PBS).

The perfluoropropane (C_3_ F_8_, Research Institute of Physical and Chemical Engineering of Nuclear Industry, Tianjin, China) was put in a hermetic bottle, and the air was withdrawn. Then, the admixture was vibrated for 45 s in a dental amalgamator (YJT Medical Apparatuses and Instruments, Shanghai, China) mechanically and sonicated the liquor with 20 kHz ultrasound probe (Sonics, USA). The nanobubbles were then suspended in PBS, cleaned with PBS and centrifuged at 800 rpm for 5 min. We collected the supernatant and collected the nanobubbles by employing the hemocytometer (Bürker-Türk, Wertheim, Germany).

The NET-1 siRNA modified with biotin was designed and synthesized from Shanghai Gene Pharma Co, Ltd. (Shanghai, China) on the basis of published NET-1 sequences from GenBank. The full length human NET-1 siRNA was as follows (duplex): Sense: 5′-GGG CAU CCU UUC UGA AGA UTT-3′, Antisense: 5′-AUC UUC AGA AAG GAU GCC CTT-3′; the sense of negative control gene was 5′-UUC UCC GAA CGU GUC ACG UTT-3′ and the antisense was 5′-ACG UGA CAC GUU CGG AGA ATT-3′.

After which, we incubated the biotinylated nanobubbles with 500 μL avidin (1 mg/mL, Sigma, USA) for 10 min at room temperature, and cleaned the nanobubbles with 3 mL PBS by centrifugation at 400 g. Finally, the nanobubbles were resuspended in 10 mL PBS for 5 min, followed by activated biotinylated Cy3-conjugated glypican-3 (GPC3; Abcam, Cambridge, MA) antibody and avidinylated nanobubbles in PBS slowly. Then the mixed liquor was stored at 4 °C overnight to form siRNA-TNBS compound. The particle size was measured by Dynamic light scattering (DLS, Zetasizer Nano ZS90, Malvern Instruments, UK) and the pattern was detected by transmission electron microscopy (TEM, Hitachi TEM system, Japan).

### Animal model

All animal experiments were approved by the Institutional Animal Care and Use Committee of Harbin Medical University Cancer Hospital and School of Life Science and Technology in Harbin Institute of Technology. All procedures were performed according to the Guidelines for Tumor Induction in Mice and Rats (Updated May 2013).

Thirty BALB/c Nude mice (female, 5–6 weeks old and weighing 18–20 g) were obtained from Beijing Vital River Laboratory Animal Technology (Charles River Laboratories, USA). A total of 5 × 10^6^ HepG2 human HCC cells (from the Institute of Cancer Research affiliated to Harbin Medical University) were suspended in 100 µL mixture containing 50 µL PBS and 50 µL BD Basement Membrane Matrix. The tumor cell suspension was then injected subcutaneously into the back skin of all the mice. The tumors were macroscopically measured as 5 × 5 mm approximately, and established the tumor-bearing nude mice models successfully. The maximum tumor diameter (MTD) of each tumor was measured by calipers and then recorded. Every tumor was measured for three to five times to decrease the measuring error.

The experiments composed of 18 nude mice, which were randomly divided into three groups, with one tumor implanted per mice. Cohort A (six mice) was treated with NET-1 siRNA TNBs; Cohort B (six mice) was treated with negative control gene TNBS; and Cohort C (six mice) was treated with saline solution. Maximum diameter of each tumor was used to evaluate tumor growth and measured with vernier valiper. The first treatment was considered as the first day of the experiment, the experiment lasted 60 days. When the maximum diameter of tumor was more than 20 mm (day 42), the experimental animal were ethical executed (three nude mice), all nude mice were executed after the experiment.

### Therapeutic process

The ability of targeting and transfection efficiency were validated according to the previous experiments (Wu et al., 2016). Treatments were administered to the model mice four times (day 1, 5, 9, and 13). Mice of cohorts A and B were injected through tail vein with a single dose (100 mg/kg) of TNBS per therapy, and cohort C was injected with saline solution. After injection, all the tumors were irradiated with low-frequency ultrasound instantly by using Low Frequency Ultrasound Transfection Instrument (Institute of Ultrasound Imaging, Second Affiliated Hospital of Chongqing Medical University, Chongqing, China) at 1 MHz frequency, yielding 50% duty cycle, 1.0 W/cm (Bruix et al., 2014) intensity, and 1 min for each tumor as duration of irradiation. During the experimental process, all the mice died naturally or culled by cervical dislocation when their tumors reached the ethical limits.

### US-SWE

US-SWE is performed three times (day 0, 10, and 21). SWE measurement and treatment were performed after the MTD of tumor over 5 mm. During SWE management and image acquisition, mice were placed on a cushion heated to 37 °C approximately to maintain animal temperature under isoflurane anesthesia (2 mL/min in 100% O_2_) (Elyas et al., 2017). An Aixplorer US system with high-frequency probe (Super Linear TM SL15-4, Super Sonic Imagine, Aix-en-Provence, France) was used. All SWE measurements were performed after conventional US by the same doctor who performed ultrasound (HT Shang) and has several years of clinical experience in performing superficial US as part of regular practice.

The US probe was placed in the US gel, and was applied to the scanning area for better coupling. For the US and SWE measurements, the picture in the transverse and sagittal plane were obtained, with three to five repetitions per time for each tumor and the median value was taken for the current measurement. The measurements were taken for three times during the experimentation. In the process of SWE measurement, we have drawn the region of interest (ROI) free to produce the result, and easily reflected the hardness of irregular tumor. The measurement scans the image of tumor hardness and converts it to kPa. Max and mean values of elastic modulus and standard deviation (SD) were recorded. An ROI including the whole tumor excluding the skin was drawn.

### Immunohistochemical analysis

Tumors were excised at the end of the experiments. The removed tissues were fixed in 10% formalin solution and then stored in alcohol solution before they were embedded into paraffin. Immunohistochemical expression was performed by a pathologist with a light microscope.

### Statistical analysis

Two-way ANOVA was used to compare the elastic modulus (Emax and Emean) changes among the three groups. Pearson’s correlation was used to compare between MTD and SWE values. *p*-Values less than 5% were considered as statistically significant. The survival rates were compared by log-rank test.

## Results

**Figure 1.**
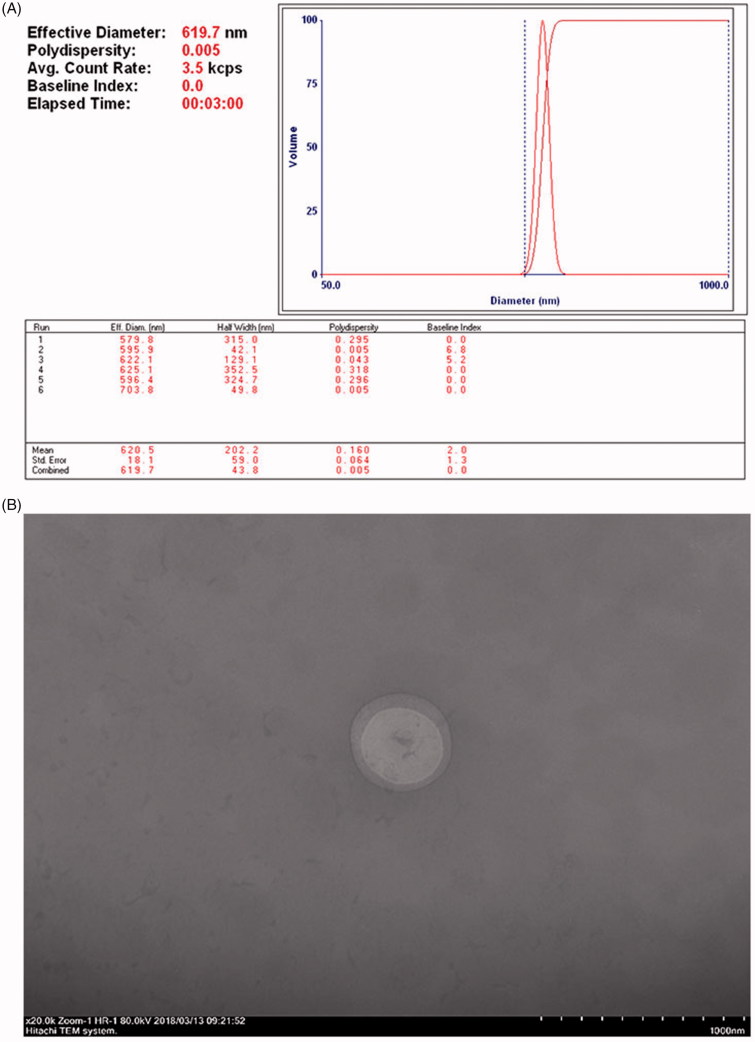
Characterization of NET-1siRNA-conjugated TNBs. (A) Size distribution of nanobubble as measured by DLS. (B) TNBs conjugated with NET-1 siRNA were observed under TEM (magnification 20k, 80 kV, scar bars: 1000 nm).

The Figure 1 showed characterization of NET-1 TNBS. The GPC3 antibody and NET-1 siRNA were conjugated to the surface of nanobubbles, and the mean diameter was 620.5 ± 18.1 nm (Figure 1(A)). The nanobubble had a smooth round surface, with an average diameter in nanoscale and an appropriate polydispersity (Figure 1(B)). A total of 30 mice were implanted with one tumor each and 2 mice died because of infection. Due to varied time of tumor growth, we selected 18 mice with similar size and roughly grouped. During SWE measurement, we collected the elastic modulus of the tumors (the maximum recorded as Emax and the mean values recorded as Emean) for statistical analysis. The specific numerical value of the whole experiment was shown in Table 1.

**Table 1.** Detailed information of the tumors and SWE measurement of each cohort.

	Cohort	SWE 1	SWE 2	SWE 3	*p* Value
Time of experiment (day)		0	10	21	
Number of survival mice (n)	A	6	6	5	
	B	6	5	4	
	C	6	5	3	
Number of tumor measured (n)	A	6	6	5	
	B	6	5	4	
	C	6	5	3	
MTD (mm)	A	5.36 (0.14)	7.47 (0.33)	8.57 (0.30)	
	B	5.35 (0.22)	10.02 (0.23)	13.62 (0.21)	
	C	5.24 (0.15)	10.21 (0.38)	13.45 (0.27)	
Emax (kPa)	A	36.55 (4.61)	45.07 (3.22)	50.92 (3.89)	.0001*
	B	35.21 (2.75)	39.86 (2.57)	44.39 (2.39)	.0005*
	C	34.40 (5.07)	39.59 (2.25)	45.77 (1.67)	.0041*
	*p* Value	.6839	.0084*	.0248*	
Emean (kPa)	A	17.59 (1.89)	21.03 (2.17)	25.50 (1.55)	<.0001*
	B	18.55 (2.47)	20.90 (1.76)	22.18 (1.72)	.0472*
	C	18.27 (4.15)	17.83 (1.48)	21.76 (1.80)	.0006*
	*p* Value	.595	.56	.0239*	

* *p* < .005.

Standard deviation values are shown in brackets.

The number of mice decrease because of accidental death and ethical executed which tumor size attain to 20 mm.

MTD: maximum tumor diameter.

Firstly, SWE values (Emax and Emean) of the tumors showed increased values over time in the three groups. Pearson’s correlation between the tumor size and SWE values for all three groups were calculated. Results showed a positive correlation between Emax and tumor size (Cohort A: *r* = 0.9978, *p* = .042; Cohort B: *r* = 0.9973, *p* = .0464; Cohort C: *r* = 0.9980, *p* = .0403), but Emean showed no significant correlation with tumor size.

**Figure 2.**
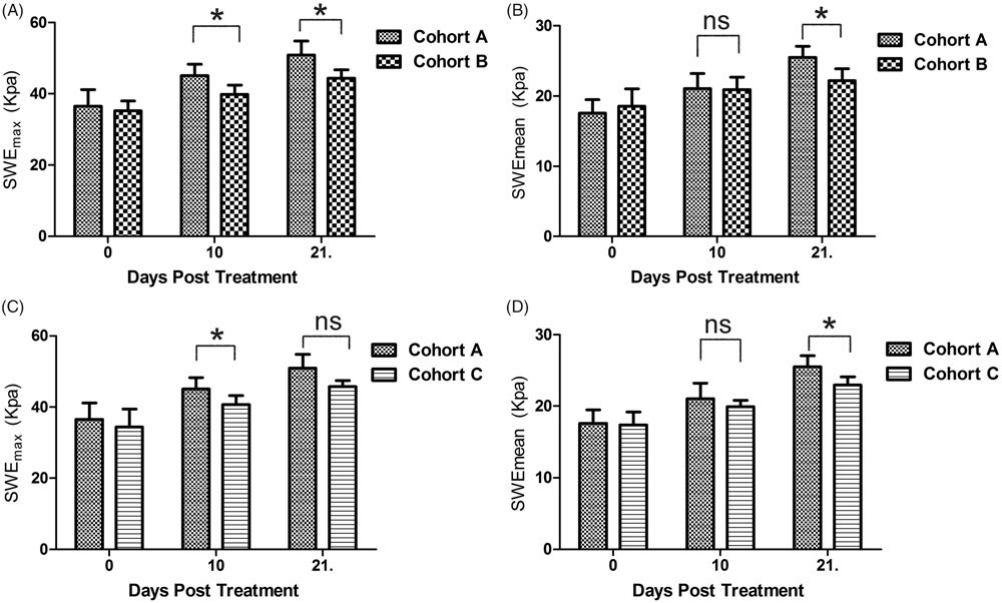
Comparison of SWEmax (A, C) and SWEmean (B, D) between Cohort A and B, Cohort A and C. *p < .05, ns: no significant.

**Figure 3.**
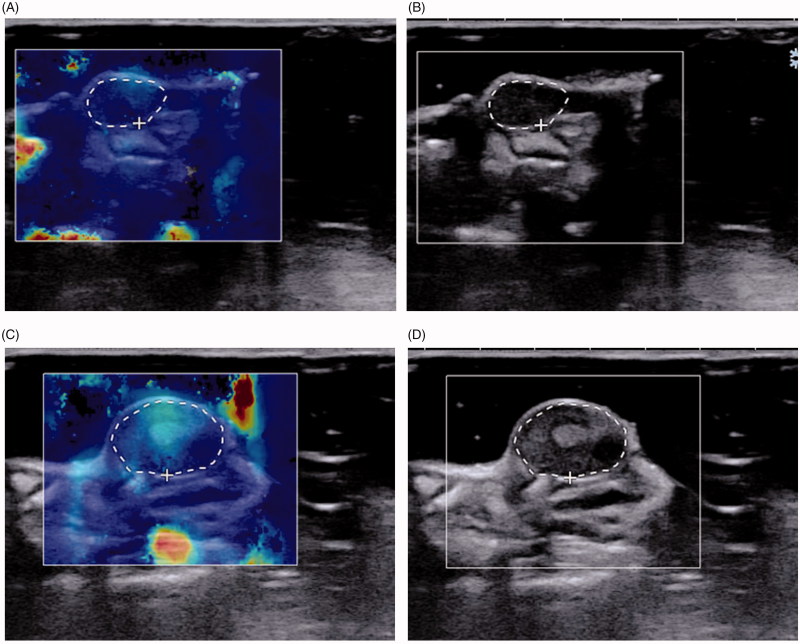
SWE images of tumor in a mice treated with NET-1 siRNA TNBs that were measured on day 0 and day 21. (A, B) (day 0) MTD:5.36 mm, Emax: 24.3 kPa, Emean: 13.6 kPa. (C, D) (day 21) MTD: 8.69 mm, Emax: 39.7 kPa, Emean: 21.0 kPa. White circles correspond to the regions of interest, which contained the whole tumor of the current section, within which the max and mean stiffness values were measured.

Secondly, Emax and Emean values showed no differences in the three groups before the first treatment (day 0). But the second (day 10) and third (day 21) SWE measurements showed differences in the Emax between the three groups, where cohort A showed increased hardness more obviously (Figure 2(A and C)). Emean of the tumors showed no statistical differences in the three groups in the first two measurements. But the Emean of cohort A was higher than cohorts B and C in the third measurement and showed statistical differences (Figure 2(B and D)). An image example was shown in Figure 3.

**Figure 4.**
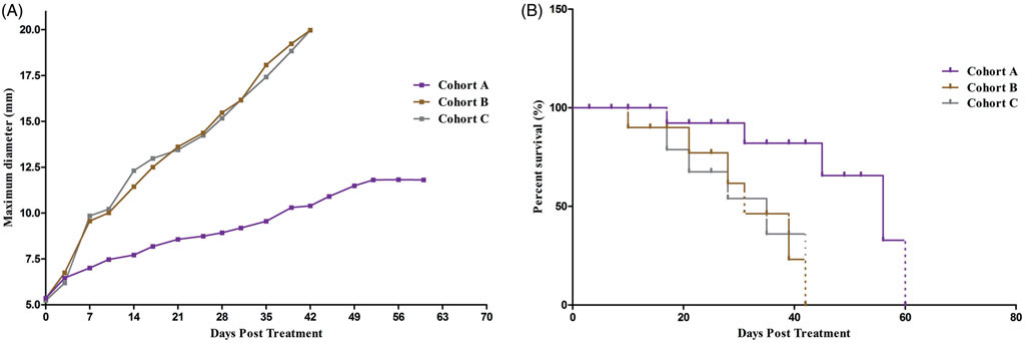
MTD in all nude mice and survival rate in the three Cohorts. MTD was increased rapidly during the first 1–2 weeks, and then slowed over time. The tumor size in Cohort A was increased slowly and the median survival time was significantly prolonged in Cohort A compared with Cohorts B and C after treatment.

The tumor growth curve and survival rates of the three groups were shown in Figure 4. Although the tumor sizes increased, the tumor growth rate in cohort A was significantly reduced compared with other groups and increased the survival rate. In the curve, we found that the tumor growth of cohort A was significantly slow than cohorts B and C (*p* < .05). Beyond the mice that naturally die during the experiment, two mice of cohorts B and C were ethically executed because the size of the tumor achieved 20 mm.

**Figure 5.**
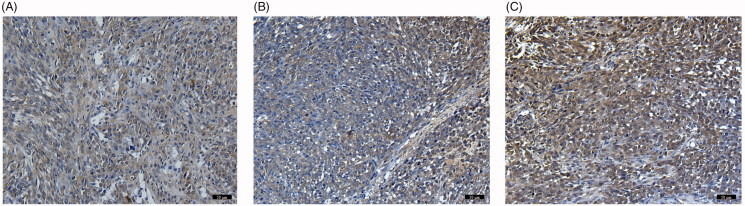
Immunohistochemical staining (magnification, ×100, scar bars: 100 µm) of Cohort A, Cohort B and Cohort C. There was no statistical difference in the level of NET-1 protein between B (Cohort B) and C (Cohort C). The NET-1 expression was significantly decreased in A (Cohort A) compared with the other two groups (*p* < .05).

Immunohistochemical results showed that the expression of NET-1 protein in tumor tissues in Cohort A was significantly lower than that in the other two groups and no significant differences were observed between the two groups (Figure 5).

## Discussion

Recently, SWE has been widely used in liver diseases (Kasai et al., 2015), and at the same time, gene therapy remained a hot research point in the treatment of liver cancer (Shanbhogue et al., 2011). In the previous studies, the treatment effect of NET-1 siRNA was always evaluated in vitro or pathologically and histological analysis after executing the animal models (He et al., 2013). Few scholars have studied the hardness of HCC tumor tissues in animal models and the changes after gene therapy. In addition, many studies have been conducted to assess the effects of SWE in *in vitro* studies, while few in the *in vivo* studies in small animals (Chamming’s et al., 2013). Our study used the noninvasive real-time SWE for the treatment process and evaluated the effect of gene therapy by analyzing the changes of tumor hardness. In addition, another innovation of our study was that the adoption of a new type of gene carrier (TNBS) to transfect NET-1 siRNA. The ability of target searching and the transfection capacity of TNBS have been verified in the preliminary experiments by our research group (Wu et al., 2016), and so it was not expounded in this paper.

In our study, we found that the Emax values of tumor stiffness were increased significantly during tumor growth, and were correlated with tumor size, but the Emean values showed no such obvious changes. Previous studies have shown that the small tumors were softer than larger ones in other types of cancer xenografts (Evans et al., 2012). HCC in humans showed lower stiffness and smaller size of the lesions (Ronot et al., 2015), which was in line with our study results. The changes in stiffness are generally because of the collagen deposition and further post-transitional modifications of extracellular matrix components during the rapid progression of cellular expansion (Wells, 2008). The proliferation of cells and the proportion of necrosis were decreased with tumor growth, and so, the tumor becomes stiff.

In this study, we found that SWE could be used to detect the changes of stiffness associated with cancer drug therapeutic effect in nude mice. After treatment, all the tumors of the three cohorts continued to grow, but the growth rate was lower in the NET-1 siRNA treated group, and this was identified using the drug therapeutic effect (Elyas et al., 2017). The trend of hardness in the three groups was similar over time, but the Emax values of the tumors in Cohort A were increased faster than others. Before the first treatment, there were no differences among the three groups. During therapy, the stiffness was increased in all the groups, and these results were different with the previous study results (Chamming's et al., 2016). The reason for this was due to specific pathologic modifications in response to the prior treatment. However, the changes of Emax in Cohort A was significant than other groups, suggesting that the NET-1 TNBS could change the structure inside the tumor, and could be detected by SWE. This phenomenon could be due to the higher cell density of the treated tumors than control groups. However, there were no definite answers for the changes of tumor stiffness after treatment, and most authors reported softening of cancers under effective therapy (Falou et al., 2013). But in our study, we found that the effect of treatment could be associated with tissue stiffening, but this was not associated with poor therapy.

In addition, the changes of Emean were not significant between the treatment group and the other two groups, attributing to the development of bigger necrotic area over time. We thought that the hardest part (Emax) of the tumor had the most meaningful value when evaluating its curative effect.

There were two novel points of our study compared with other similar studies. Firstly, the study used TNBS as a carrier of gene transfection, which could induce tumor cells to generate gene products that kill cancer cells without damaging the healthy tissue (Caskey et al., 2011), and the combination of TNBS and low-frequency US exposure have distinct potential to improve the gene transfection efficiency (Hong and Nam, 2014). Secondly, the shape of ROI in SWE measurement was irregular, ensuring that all the parts of the tumors were measured in the measuring section.

This study established the subcutaneous tumor model which is the most commonly used in animal experiments, the tumor tissue intact the characteristics of human HCC, and more intuitive than the orthotopic models and objective evaluation of curative effect, benefits include building a high success rate, short building time, size of the tumors can real-time measurement, easier to SWE. But the downside is the lack of the interaction between the tumor and liver tissue reaction, and possible false positive results, also may be due to the different environment affect the biological behavior of malignant cells (Lu et al., 2007). Orthotopic tumors have certain advantages in mimicking the microenvironment of human liver cancer, but they are not used because the tumor volume cannot be measured in real time (Yoo et al., 2019).

Our study has several limitations. Firstly, we did not discuss the specific effect of necrosis to the SWE values, because the SWE measurement was performed in the early stage of the experiment. Secondly, the number of animals was smaller and the undetailed grouping, such as TNB with no gene was not included, this is due to the experiment is only a preliminary exploration and study on this aspect, we will increase the number of sample of experiment, replace the animal models and build liver carcinoma model in situ in subsequent studies, at the same time, the experimental group will use a variety of elastography measurement methods to assess the change of tumor hardness. Therefore, more accurate data will be obtained to support the above experimental results. In conclusion, we found that the TNBS conjugated with NET-1 siRNA could affect the growth of the tumor, and SWE provided a noninvasive and real-time imaging method to detect the potential changes in the tumor development.

## References

[CIT0001] Baruteau J, Waddington SN, Alexander IE, et al. (2017). Gene therapy for monogenic liver diseases: clinical successes, current challenges and future prospects. J Inherit Metab Dis 40:497–517. 28567541 10.1007/s10545-017-0053-3 PMC5500673

[CIT0002] Bruix J, Gores GJ, Mazzaferro V (2014). Hepatocellular carcinoma: clinical frontiers and perspectives. Gut 63:844–55. 24531850 10.1136/gutjnl-2013-306627 PMC4337888

[CIT0003] Caskey CF, Hu X, Ferrara KW (2011). Leveraging the power of ultrasound for therapeutic design and optimization. J Control Release 156:297–306. 21835212 10.1016/j.jconrel.2011.07.032 PMC3243361

[CIT0004] Chamming’s F, Latorre-Ossa H, Le Frère-Belda MA, et al. (2013). Shear wave elastography of tumour growth in a human breast cancer model with pathological correlation. Eur Radiol 23:2079–86. 23553589 10.1007/s00330-013-2828-8

[CIT0005] Chamming’s F, Le-Frère-Belda M-A, Latorre-Ossa H, et al. (2016). Supersonic shear wave elastography of response to anti-cancer therapy in a xenograft tumor model. Ultrasound Med Biol 42:924–30. 26746382 10.1016/j.ultrasmedbio.2015.12.001

[CIT0006] Chen L, Wang Z, Zhan X, et al. (2007). Association of net-1 gene expression with human hepatocellular carcinoma. Int J Surg Pathol 15:346–53. 17913940 10.1177/1066896907306083

[CIT0007] Das SK, Menezes ME, Bhatia S, et al. (2015). Gene therapies for cancer: strategies, challenges and successes. J Cell Physiol 230:259–71. 25196387 10.1002/jcp.24791 PMC4363073

[CIT0008] Davidson BL, Mccray PB. Jr.(2011). Current prospects for RNA interference-based therapies. Nat Rev Genet 12:329–40. 21499294 10.1038/nrg2968 PMC7097665

[CIT0009] El Kaffas A, Bekah D, Rui M, et al. (2013). Investigating longitudinal changes in the mechanical properties of mcf-7 cells exposed to paclitaxol using particle tracking microrheology. Phys Med Biol 58:923–36. 23340402 10.1088/0031-9155/58/4/923

[CIT0010] Elyas E, Papaevangelou E, Alles EJ, et al. (2017). Correlation of ultrasound shear wave elastography with pathological analysis in a xenografic tumour model. Sci Rep 7:165. 28279018 10.1038/s41598-017-00144-5 PMC5427848

[CIT0011] Evans A, Whelehan P, Thomson K, et al. (2012). Invasive breast cancer: relationship between shear-wave elastographic findings and histologic prognostic factors. Radiology 263:673–7. 22523322 10.1148/radiol.12111317

[CIT0012] Falou O, Sadeghi-Naini A, Prematilake S, et al. (2013). Evaluation of neoadjuvant chemotherapy response in women with locally advanced breast cancer using ultrasound elastography. Transl Oncol 6:17–24. 23418613 10.1593/tlo.12412 PMC3573650

[CIT0013] Ferrara K, Pollard R, Borden M (2007). Ultrasound microbubble contrast agents: fundamentals and application to gene and drug delivery. Annu Rev Biomed Eng 9:415–47. 17651012 10.1146/annurev.bioeng.8.061505.095852

[CIT0014] Forner A, Gilabert M, Bruix J, et al. (2014). Treatment of intermediate-stage hepatocellular carcinoma. Nat Rev Clin Oncol 11:525–35. 25091611 10.1038/nrclinonc.2014.122

[CIT0015] Geis NA, Katus HA, Bekeredjian R (2012). Microbubbles as a vehicle for gene and drug delivery: current clinical implications and future perspectives. Curr Pharm Des 18:2166–83. 22352771 10.2174/138161212800099946

[CIT0016] He S, Wei YZ, Wang GL, et al. (2013). Study of RNA interference targeting net-1 combination with sorafenib for hepatocellular carcinoma therapy in vitro and in vivo. Gastroenterol Res Pract 2013:685150. 24307893 10.1155/2013/685150 PMC3838818

[CIT0017] Hong CA, Nam YS (2014). Functional nanostructures for effective delivery of small interfering RNA therapeutics. Theranostics 4:1211–32. 25285170 10.7150/thno.8491 PMC4183999

[CIT0018] Kasai Y, Moriyasu F, Saito K, et al. (2015). Value of shear wave elastography for predicting hepatocellular carcinoma and esophagogastric varices in patients with chronic liver disease. J Med Ultrason (2001) 42:349–55. 26576786 10.1007/s10396-014-0603-3

[CIT0019] Kumar SR, Markusic DM, Biswas M, et al. (2016). Clinical development of gene therapy: results and lessons from recent successes. Mol Ther Methods Clin Dev 3:16034. 27257611 10.1038/mtm.2016.34 PMC4879992

[CIT0020] Li J, Jamin Y, Boult JK, et al. (2014). Tumour biomechanical response to the vascular disrupting agent zd6126 in vivo assessed by magnetic resonance elastography. Br J Cancer 110:1727–32. 24569471 10.1038/bjc.2014.76 PMC3974089

[CIT0021] Li T, Xue Y, Wang G, et al. (2016). Multi-target sirna: therapeutic strategy for hepatocellular carcinoma. J Cancer 7:1317–27. 27390607 10.7150/jca.15157 PMC4934040

[CIT0022] Lu YS, Kashida Y, Kulp SK, et al. (2007). Efficacy of a novel histone deacetylase inhibitor in murine models of hepatocellular carcinoma. Hepatology (Baltimore, Md) 46:1119–30. 10.1002/hep.21804 17654699

[CIT0023] Piscaglia F, Marinelli S, Bota S, et al. (2014). The role of ultrasound elastographic techniques in chronic liver disease: current status and future perspectives. Eur J Radiol 83:450–5. 23891139 10.1016/j.ejrad.2013.06.009

[CIT0024] Ronot M, Di Renzo S, Gregoli B, et al. (2015). Characterization of fortuitously discovered focal liver lesions: additional information provided by shearwave elastography. Eur Radiol 25:346–58. 25231131 10.1007/s00330-014-3370-z

[CIT0025] Shanbhogue AK, Prasad SR, Takahashi N, et al. (2011). Recent advances in cytogenetics and molecular biology of adult hepatocellular tumors: Implications for imaging and management. Radiology 258:673–93. 21339346 10.1148/radiol.10100376

[CIT0026] Wells RG (2008). The role of matrix stiffness in regulating cell behavior. Hepatology 47:1394–400. 18307210 10.1002/hep.22193

[CIT0027] Wu B, Qiao Q, Han X, et al. (2016). Targeted nanobubbles in low-frequency ultrasound-mediated gene transfection and growth inhibition of hepatocellular carcinoma cells. Tumor Biol 37:12113–21. 10.1007/s13277-016-5082-2 27216880

[CIT0028] Yoo JJ, Yu SJ, Na J, et al. (2019). Hexokinase-ii inhibition synergistically augments the anti-tumor efficacy of sorafenib in hepatocellular carcinoma. Int J Mol Sci 20:pii: E1292. 10.3390/ijms20061292 PMC6471302 30875800

[CIT0029] Zhang J, Wang J, Chen L, et al. (2014). Expression and function of net-1 in human skin squamous cell carcinoma. Arch Dermatol Res 306:385–97. 24196235 10.1007/s00403-013-1423-9 PMC4000423

